# An Evaluation of Relative Damage to the Powertrain System in Tracked Vehicles

**DOI:** 10.3390/s90301845

**Published:** 2009-03-13

**Authors:** Sang-Ho Lee, Jeong-Hwan Lee, Sang-Hwa Goo, Yong-Cheol Cho, Ho-Young Cho

**Affiliations:** Instrumentation and Data Analysis Group, Chang-won Proving Ground, Fifth Technology Research Center, Agency for Defense Development/P.O.B.126 Changwon, Gyungnam, Korea

**Keywords:** Relative damage, driving duty, revolution counting, fatigue life

## Abstract

The objective of this study was to improve the reliability of the endurance test for the powertrain system of military tracked vehicles. The measurement system that measures the driving duty applied to the powertrain system caused by mobility on roads consists of eight analog channels and two pulse channels, including the propeller shaft output torques for the left and right sides. The data obtained from this measurement system can be used to introduce a new technology that produces the output torque of a torque converter and that can be applied to analyze the revolution counting for the endurance and road mobility in the front unit and represent the relative fatigue damages analysis technique and its results according to the driven roads through a cumulative fatigue method.

## 1. Introduction

The main factors affecting the endurance life of tracked vehicle mobility systems are the vibrational environment caused by road profiles, the obliqueness of roads, frequency of oblique roads, oblique continuity, and left and right steering characteristics.

The vibration environment caused by the road profile mainly affects the suspension and structure of vehicle. The severity for test courses at the Changwon proving ground and the mobility roads in army operation areas (AOAs) was measured and compared using the profilometer which was developed by the Changwon proving ground in the Agency for Defense Development [1]. Through the comparison of analyzed severity, the standardization of severity for test courses at the Changwon proving ground was achieved [2].

The characteristics of the powertrain system of mobility systems have a close relation to the obliqueness of roads, frequency of oblique roads, oblique continuity, and left and right steering characteristics. Studies related to the durability of powertrain systems have been performed partially by commercial vehicle manufacturers. Kakinoki performed a study for life prediction of turbine-generator shafts which the rotational components like those found in powertrain systems [3]. Tohru and Masanori established an accelerated test method corresponding with the severity of service conditions to perform endurance tests on auto transmissions, and evaluated the durability characteristics of auto transmissions in the laboratory or proving ground [4–5]. Kim and Shin studied a computer simulation method regarding the cumulative damage prediction for the gears and bearings of auto transmissions [6–7], and Kong calculated and compared the damage of rotational components using both rainflow counting and revolution counting [8].

But, although needs for the investigation of the comparison based on the duty levels between the Changwon proving ground in the Agency for Defense Development that exclusively charges the endurance test of military mobility vehicles and the mobility roads in army operation areas (AOAs) and the study on the correlation according to the characteristics of applied duties have been required. However, the needs have not yet been attempted.

The goal of this study was to verify whether the duty levels and characteristics of the powertrain system for the endurance test ground present the proper conditions and characteristics as endurance test grounds through the comparison and analysis of the endurance test and mobility roads at the front unit. It can be regarded that the evaluation of the driving duty levels and characteristics for the endurance test ground in mobility test fields and mobility roads in army operation areas includes not only the technical test for the powertrain system, but also the test condition that is to be verified in order to guarantee the reliability of the endurance test that affects the powertrain system.

A previous study tried to achieve this goal based on wheeled vehicles. In the case of wheeled vehicles, the same analysis technique can be applied to military trucks and jeeps that represent similar powertrain system mechanisms but there are limitations in the direct application to tracked vehicles due to the differences in powertrain mechanisms, such as vehicle specifications, engine and transmission characteristics, and driving styles [1–2].

This study measured the driving duties presented on a paved course, gravels, and a cross country course established at the Changwon Proving Ground for the mass-produced 000 tracked vehicle. This study also quantified the relative fatigue damages for the major elements of the powertrain system by applying Revolution Counting, Miner’s Rule, and cumulative fatigue damage theory using the torque converter output torque and engine rpm produced during the driving on the corresponding road based on the measurement results.

As a result, it provides important data for considering the endurance duties, including the guarantee of the self-design technique in the endurance test ground of powertrain systems and basic technique, for shortening the endurance test of the powertrain system, which reflects the duty characteristics of the mobility roads in army operation areas.

## 2. Measurement System and Results

### 2.1. Configuration of the Measurement System

Although the measurement of engine output torque is the most effective way to measure the load of a powertrain system, the measurement environmental conditions, such as high temperature and lack of space for the measurement, are generally insufficient. Thus, this study applied a method that calculates the torque converter output torque using gear ratios by measuring the output torque on a propeller shaft in order to perform an analysis of relative duties. This method was also proposed by CARLOS, which is a European standard load cooperation research association and that used the torque converter output torque for an automatic transmission and engine output torque for a manual transmission.

**Figure 1.**
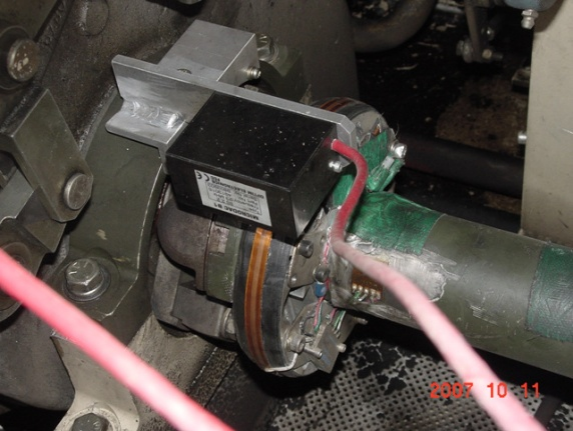
Torque sensor for the propeller shaft.

**Figure 2.**
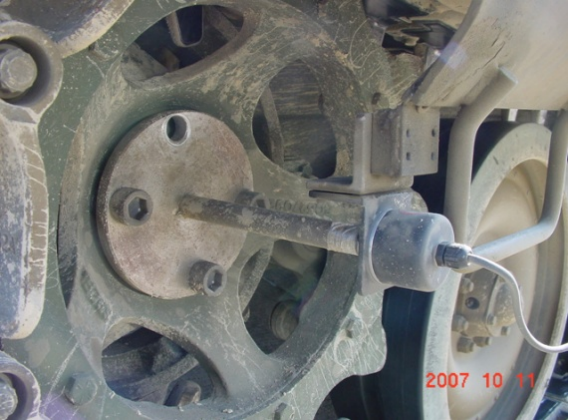
Speed sensor for the final drive.

As shown in Figure 1, a near field telemetry device was used to measure the output torque on a propeller shaft in which a strain gauge with a 350 ohm full bridge was used as a torque sensor. Because tracked vehicles use a control method that divides the torque converter output torque into transmission and steering input torques, it is necessary to measure the speed of the final drive for calculating the torque converter output torque from the propeller shaft torque that should be calculated by dividing it into the existence of steering, transmission output torque (or ring gear torque), and steering torque (or sun gear torque). Figure 2 illustrates the installed encoder using the tool fabricated in this study.

**Figure 3.**
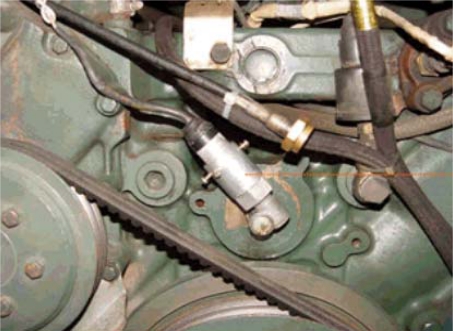
Tacho-generator for the engine RPM measurement.

Engine rpms represent the degree of the duty applied to the engine during a vehicle’s operation and they are important data to calculate relative duties. To measure the engine rpm, a tacho-generator that generates voltages in proportion to the engine rpm in a vehicle, as shown in Figure 3, was used. In addition, the vehicle speed was measured using a VBOXII sensor with a GPS that consists of an antenna for receiving GPS signals and body and has the resolution of 20 km/h/V.

The real-time transmission shifting stages during driving represent important information, not only about the duty level of the transmission on the measured road, operation distribution for each shifting stage, and shifting frequency, but also the reverse calculation of the torque converter output torque in a relative duties calculation process. The shifting stage applied to the 000 tracked vehicle used a method that measures the shifting stages by measuring the pressure applied to a clutch solenoid for gear shifting generated in a shifting stage.

### 2.2. Results

In general, it is necessary to consider a reference for the speed due to the significant change in the torque characteristics in actual powertrain systems, according to changes in vehicle velocities. In the civilian car industries, the vehicle speed that is determined by 90% of regular drivers is considered as a measurement speed for its corresponding roads. Regarding the speed configuration for measuring the driving duty in the endurance test ground, this study configured the average driving speed presented in the previously performed endurance testing of tracked vehicles as a measurement speed for the subjective road.

**Figure 4.**
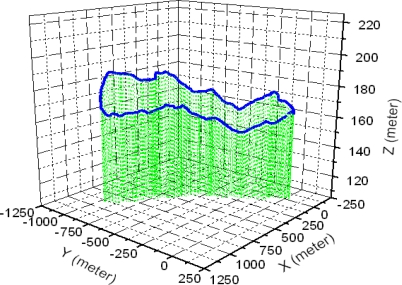
3D profile for the cross-country course.

**Figure 7.**
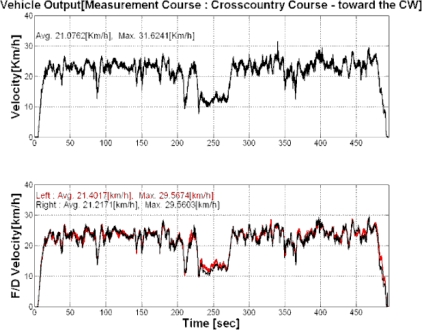
Vehicle speed and final drive speed of the cross-country course.

This study performed the measurement for three types of endurance test grounds and three mobility roads in army operation areas. In addition, the cross country course was configured as a loop type of road with about 3.0 km of length and 8 m of width as shown in Figure 4. The conditions of the cross country course represented not only severe bends, but also various periods in these bends. The average driving speed in this test road was about 20 km/h. Figured 5 and 6 show the output torque for the left and right driving shafts, engine rpms, and shifting stages in which the major shifts were performed at the second and third gear stages. In addition, it can be seen that the average measurement speed obtained was 21.1 km/h, as shown in Figure 7, and similar to the target speed of 20 km/h.

Furthermore, the same measurements and calculations were applied to the linear pavement and gravel roads, and the results are presented in Table 1. Although the pavement showed the highest values for the engine rpm and vehicle speed, the highest torque of the propeller shaft generated by the gear shifting stages ratio was recorded on the cross country course. Table 2 represents the results of the mobility roads of Y, J, and K roads in army operation areas where the lengths of the maneuvering roads were determined as 1.0 km, 1.6 km, and 9.7 km, respectively. The vehicle speed and engine rpm showed the highest values in the K area as 21.7 km/h and 1,414, respectively. Whereas, as noted in Table 1, the highest torque of the propeller shaft generated by the ratio of gear shifting stages was presented in the Y area.

## 3. Analysis of Relative Fatigue Damages

### 3.1. Torque Converter Output Torque

#### 3.1.1 Theoretical Background

**Figure 8.**
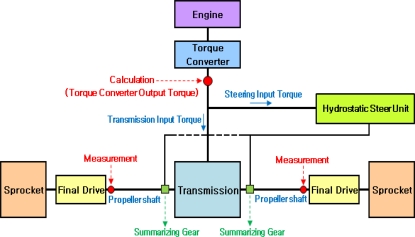
Configuration of the torque transmission for the tracked vehicle.

In the case of the tracked vehicle like the 000 tracked vehicle, the power generated from the engine will pass through a torque converter, and a part of the power generated from the torque converter turbine will be discharged to operate pans, intake valves, and exhaust pumps. Then, the remaining power will pass through input gears in order to establish their operation and steering. The input gear transfers the power to the transmission for operating the hydro-static steering and driving systems. The power input to the hydro-static pump is also used as power for the steering, but the input driven gear uses it as the input torque of the transmission for the driving system. The output torque (ring gear torque) from the transmission and the steering torque (sun gear torque) are to be combined at a planetary gear (summarizing gear) in which the combined torque is transferred to the propeller shaft on the the left and right sides and that is transferred to the driving wheels via a final reduction gear as seen in Figure 8. As mentioned previously, because tracked vehicles draw the power for driving and steering from the torque converter, differently than wheeled vehicles, it is not possible to calculate the torque converter output torque only using a linear relationship, such as gear ratios, from the output torque of the propeller shaft, and it is therefore necessary to separate the output torque of the propeller shaft into ring and sun gear torques.

The relationship between the gear ratio, the rpm of the summarizing gear, which is a type of planetary gear, and the torque can be expressed by Equation (1) as follows:

(1) $$T_{C}=T_{R}+T_{S},N_{C}=\frac{Z_{R}N_{R}+Z_{S}N_{S}}{Z_{R}+Z_{S}}$$

where, N_C_ is the number of rotations, N_R_ and N_S_ show the number of rotations in ring and sun gears, respectively, Z_R_ and Z_S_ show the number of teeth in ring and sun gears, respectively, and T_C_, T_R_, and T_S_ represent the torque of the propeller shaft, ring gear, and sun gear, respectively.

In addition, because the torque is distributed to the left and right ring gears at the output of the transmission as a 50:50 ratio, it can be denoted as in Equation (2). Also, the torques generated from the hydro-static pump and motor are transferred to the sun gear of the summarizing gear where the torques determined at the left and right sun gears represent the same scale and reverse direction. Thus, it can be expressed as Equation (3):

(2) $$T_{R,\text{Left}}=T_{R,\text{Right}},N_{R,\text{Left}}=N_{R,\text{Right}}$$

(3) $$T_{S,\text{Left}}=T_{S,\text{Right}},N_{S,\text{Left}}=N_{s,\text{Right}}$$

By considering these equations and the gear ratio applied to the powertrain system of the 000 tracked vehicle, the input torques of the steering and driving sections (input torques of the transmission) can be denoted as in Equation (4) and Equation (5):

(4) $$T_{\text{Steering},\text{in}}=2\alpha \times \left[|T_{S,\text{Left}}|\cdot \left(\frac{34}{14}\right)\cdot \left(\frac{22}{37}\right)\right]\times \left(\frac{13}{39}\right)\cdot (\frac{34}{32})$$

(5) $$T_{\text{Driving},\text{in}}=2\times [\frac{T_{R,\text{Left}\cdot \frac{27}{56}}}{{\text{TM}}_{\text{Gear Ratio}}}]\cdot \frac{74}{69}$$

where α is the efficiency of the hydro-static pump and motor and is applied by the average of 83%. Also, the final reduction gear and gear ratios are presented in Table 3.

In addition, the torque converter output torque can be obtained as in Equation (6) by summing the input torques of the steering and driving sections and considering the gear ration of bevel gears:

(6) $$\begin{array}{l} R_{\text{TCout}}=[T_{\text{Steering},\text{in}}+T_{\text{Driving},\text{in}}]\cdot \frac{31}{20}\\ \;\;\;\;\;\;\;\;\;\;\;\;\;\;\;\;\;\;\;\;\;\;\;\;\;\;=2\alpha \times \left[|T_{S,\text{Left}}|\cdot \left(\frac{34}{14}\right)\cdot \left(\frac{22}{37}\right)\right]\times \left(\frac{13}{39}\right)\cdot \left(\frac{34}{32}\right)\cdot \left(\frac{31}{20}\right)+2\times [\frac{T_{R,\text{Left}\cdot \frac{27}{56}}}{{\text{TM}}_{\text{Gear Ratio}}}]\cdot \frac{74}{59}\cdot \frac{31}{20} \end{array}$$

#### 3.1.2 Results of the Calculation of the Torque Converter Output Torque

**Figure 9.**
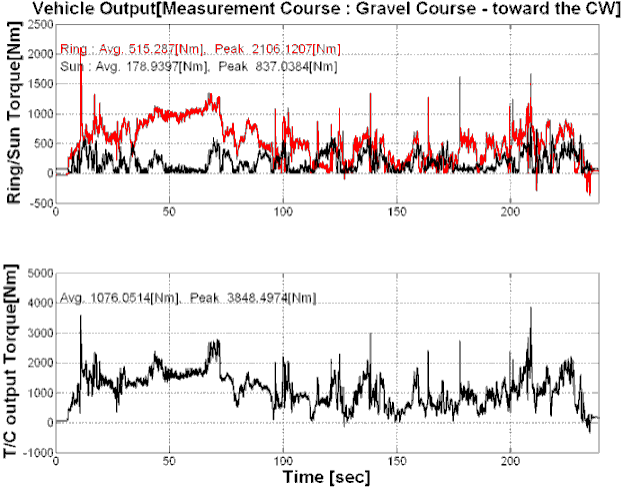
Output torque of the torque converter for the gravel course.

As mentioned above, the ring gear torque, sun gear torque, and torque converter output torque were calculated based on the results of the measurements of three endurance test and mobility roads in army operation areas. Figure 9 shows the results of the measurement on gravel, and Table 4 shows the results of the calculations of the endurance test and three mobility roads.

The ring torque and torque converter output torque showed the similar levels as that of the Y and K areas compared to that of the propeller shaft torque in which the gravels in these endurance test grounds represented the highest levels as 515.3Nm and 1,076.1Nm. It can be verified that the propeller shaft output torque was generated after the gear shifting section, and the torque converter output torque that is input before the transmission through the actual engine would be significantly varied.

### 3.2. Analysis of the Revolution Counting

It is necessary to select a proper signal classification for the goal of a target analysis in order to reduce the complex load history measured by a practical way as a simple load history for calculating the cumulative fatigue damages of machines. This classification shows some advantages that effectively reduce the huge amount of data and characterize the tendency of fatigues at the same time.

The Rainflow Counting method that is a representative method of the Cycle Counting method includes the amplitude and range of duties only, but the Revolution Counting method considers the duration time generated by specific duties and revolution speeds. It is a way that counts the number of appearances of the two signals, which are presented at each level of the predetermined finite division.

As presented in this study, it has been known that the physical quantity like a driving system in rotation generates certain fatigue damages to the parts according to the number of revolutions that corresponds to the torque application time differed from the loads generated in structures and suspension systems. This study applied the software of GlyphWorks by nCode [9].

**Figure 10.**
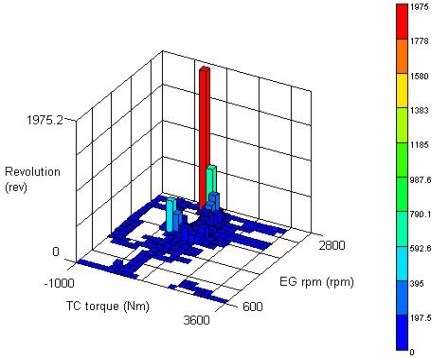
3D revolution counting for the paved course.

The level intervals (Bin Size) for performing the analysis were configured as the range of −1,000∼3,600 Nm for the torque converter output torque in which the interval was determined as 200 Nm by dividing the range into 23 sections. In the case of the engine rpm, the range of 600∼2,800 rpm was determined as 22 sections with the interval of 100 rpm. It shows that the number of revolutions is counted according to the torque converter output torque level. Figure 10 represents the results of the counting for the linear way and clockwise direction in which it can be seen that most of operations were performed at about 500 Nm of the torque converter output torque and 2800 engine rpm.

**Figure 11.**
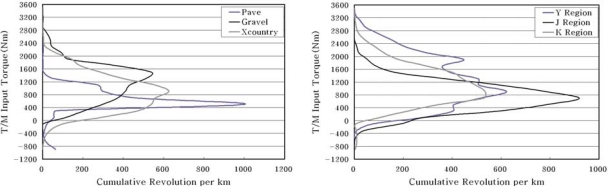
(a) Spectrum of the CPG courses. (b) spectrum of the AOA road.

Figure 11(a) illustrates the results of the normalized 2D counting with a unit km based on the results of the counting for three endurance test grounds. In the comparison of the major torque converter output torques, the cross country course was presented at about 800 Nm, and the gravels showed about 1,300 Nm that is a higher level than the cross country course relatively. Also, although these two roads showed similar levels in the cumulative number of revolutions, the gravels represented a large number of revolutions compared to that of the cross country course in a high torque range. It can be considered that certain oblique characteristics in the gravels were reflected to the results compared to that of the cross country course.

In the case of the linear endurance test ground, the major operation torque range was a low level, such as 500 Nm. It was due to the fact that the torque generated from the engine showed a low level relatively because most of driving was performed by the fourth stage of the gear at the planar road. In addition, the peak value was presented at about 1000 Nm due to the steering at turning sections.

Figure 11(b) shows the results of the 2D counting with a unit km based on the results of the counting for the mobility roads in army operation areas. It was possible to verify that the J and K areas except for the Y area showed a type of normal distribution in general.

### 3.3. Analysys of Relative Fatigue Damages

The relative fatigue damages in parts can be determined based on the data analyzed by the mentioned cycle counting method. In the estimation of the cumulative fatigue damage, this study applied a stress-service life method by modifying a torque-service life (T-N) method for applying it to rotation bodies. Also, the quantization of the cumulative fatigue damages in the parts of the powertrain system for the road surface was applied using the Miner's Rule. Thus, the relative cumulative fatigue damage, QD, can be expressed as Equation (7) [6,10–11]:

(7) $$\text{QD}=\sum_{i=1}^{n}n_{i}\cdot T_{i}^{\text{NN}}$$

where m is presented in Table 5 and expressed by $-\frac{1}{b}$ where b is a fatigue strength index, T_i_ and n_i_ represent the torque converter output torque and engine rpm, respectively, counted by the Revolution Counting method. Then, the relative fatigue damages between the endurance test grounds were analyzed by configuring the gravels as a reference damage through the mentioned process.

**Figure 12.**
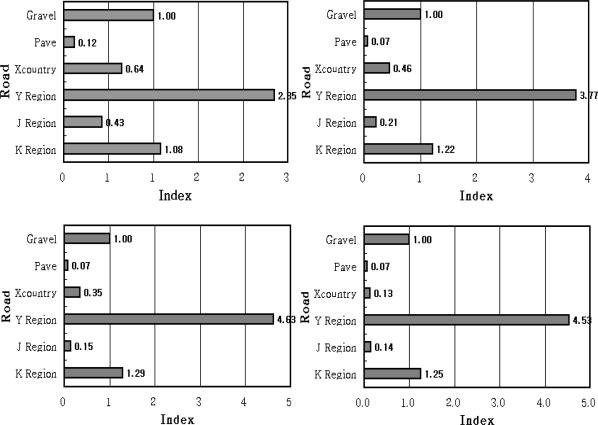
Comparision of the Quantified Damage ; (a) m = 3.33 (b) m = 6.3 (c) m = 8.389 (d) m = 17.857.

Figures 12(a) 12(b) show the results obtained from the parts of the bearing and ring gear bending in the powertrain system where the mobility roads in the K area showed the most similar results as that of the gravels. However, the mobility roads in the J area represented lower values in the torque converter output torque and cumulative engine rpm than that of the cross country course. The mobility roads in the Y area showed 2∼3 times higher relative fatigue damages than that of the gravels due to the steep slope.

Figures 12(c) and 12(d) illustrate the parts of the transmission gear bending and case in the powertrain system where the mobility roads in the K area showed m=3.33 and 6.3 as the similar values as that of the gravels. Also, the mobility roads in the J area showed a slightly lower level than that of the values of m = 3.33 and 6.3 and that demonstrated a tendency that reduces the amplitude of fatigue damages. However, it can be verified that the values significantly affected the fatigue damages as the same as the wheeled vehicles according to the major parts due to that fact that the mobility roads in the Y area demonstrated about 4.5 times higher fatigue damages than that of the gravels.

Based on these results, the results of this study can be summarized as follows: There exist large differences in the fatigue levels according to the parts of the powertrain system due to the torque converter output torque and engine rpm even though these values are obtained from the same road driving. In addition, it was evident that the intermittence rotation in shafts under a high torque state more significantly affected the fatigue damages than that of the frequent rotation in shafts under a low torque level.

**Figure 13.**
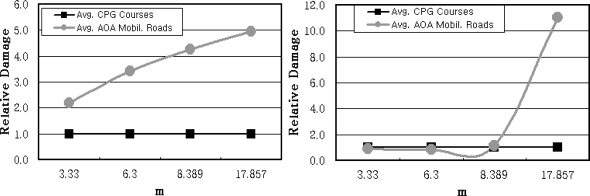
Comparison of the endurance courses and AOA roads: (a) Tracked vehicle. (b) Wheeled vehicle.

This means that it is necessary to evaluate the relative fatigue damages for the major parts of the powertrain system as a unit of each individual part even though the evaluation is applied to the duties applied to the mobility roads in an aspect of the road surface. Figure 13(a) shows the results of the comparison between the average value of the relative fatigue damages for the endurance test grounds based on the tracked vehicle and the average value obtained in three mobility roads in army operation areas. Differing from the wheeled vehicle as illustrated in Figure 13(b), most of the major parts showed low relative fatigue damages in the endurance test ground. It shows that it is necessary to additionally design and build endurance test grounds for improving the duty conditions as a large scale in order to perform the reliable durability test of the powertrain system in tracked vehicles.

## 4. Conclusions

The objective of this study was to evaluate the relative fatigue damages for specific roads by quantizing the duties of the powertrain system of the 000 tracked vehicle in the aspect of mobility roads. Thus, this study proposed a technical method that configures a measurement system and calculates the damages. Then, this study compared the results obtained this background through quantizing the duties of the powertrain system for specific endurance test grounds.

1. This study calculated the input torques of the transmission and steering by following the power transmission mechanism based on the driving shaft output torque and shifting stage. Also, this study proposed a method that quantizes the relative fatigue damages for the variable history loads based on the torque converter output torque and engine rpm.
2. This study obtained certain endurance test ground characteristics through the torque converter output torque normalized by a unit km and the analysis of cumulative revolutions.
  ♦ The gravels showed a higher level in the torque distribution compared to that of the cross country course and linear way.
  ♦ In the case of the linear way, it represented two peaks instead of presenting a normal distribution compared to that of the cross country course.
  ♦ It was verified that the mobility roads in the K and J areas showed normal distributions in a 2D spectrum analysis.
  ♦ The mobility roads in the Y area demonstrated the highest fatigue damage level.
3. By varying the gradient, m, of the T-N curve for each part in the powertrain system, the following characteristics were obtained by comparing it with the relative fatigue damages that affect the each individual part of the powertrain system.
  ♦ In the case of the K200A1, the duty levels were determined by the order of the mobility roads in the Y area > the mobility roads in the K area > the gravels > the cross country course > the mobility roads in the J area > the linear way. Also, the duty levels were quantized.
  ♦ Based on the gravels, the linear way represented almost same duty levels regardless of the values of m. However, the cross country course showed a decrease in the duties as a linear manner according to the increase in the values of m.

Furthermore, it can be seen that it is necessary to consider the duties of the powertrain system in the driving plan of the endurance test ground and perform an evaluation for each individual part in the estimation of the fatigue service life in test vehicles.

## Figures and Tables

**Figure 5.**
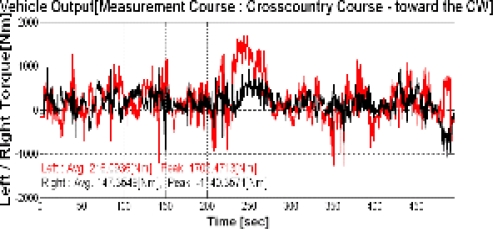
Propeller shaft torque of the cross-country course.

**Figure 6.**
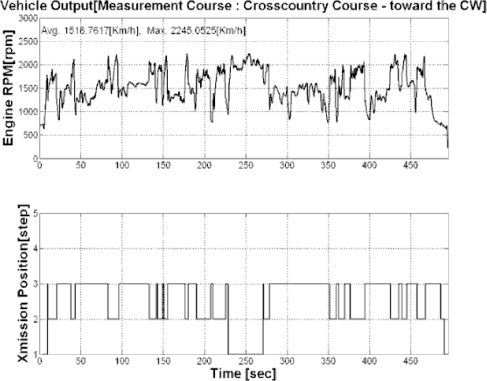
Engine RPM and transmission Level of the cross-country course.

**Table 1.** Duty characteristics for the CPG’s course.

**Factors**		**Paved**	**Gravel**	**Cross-country**

Avg. Velocity(km/h)		48.5	25.6	21.1
Transmission step		3/4	2/3	2/3
Avg. Engine RPM		2,014	1,595	1,516

Avg. Propeller shaft	Left	49.0	26.0	21.4
Velocity (km/h)	Right	49.0	25.7	21.2

Avg. Propeller shaft	Left	33.9	196.0	216.1
Torque (Nm)	Right	128.9	131.7	147.4

**Table 2.** Duty characteristics for the AOA’s road.

**Factors**		**Paved**	**Gravel**	**Cross-country**

Avg. Velocity (km/h)		14.9	16.6	21.7
Transmission step		2	2	2/3
Avg. Engine RPM		1,343	1,398	1,414

Avg. Propeller shaft	Left	15.5	17.0	22.2
Velocity(km/h)	Right	15.4	16.9	22.1

Avg. Propeller shaft	Left	417.9	226.7	216.0
Torque (Nm)	Right	319.3	202.4	200.8

**Table 3.** Gear Ratio of the Final drive and Transmission for the 000 armor.

**Gear** **Final drive**		Ratio 3.929

**Transmission**	**1-step**	3.0679
	**2-step**	1.7284
	**3-step**	1.0794
	**4-step**	0.7670
	**Rear step**	4.8807

**Table 4.** Comparison of the Estimated Torque Results

	**Avg. Ring Torque(Nm)**	**Avg. Sun Torque(Nm)**	**Avg. Torque Converter Output Torque(Nm)**

**Paved**	257.5	58.8	490.2
**Gravel**	515.3	178.9	1,076.1
**Cross-country**	380.9	140.2	807.7
**Y Region**	563.4	155.1	1,113.6
**J Region**	339.4	112.6	700.6
**K Region**	565.2	149.5	1,107.7

**Table 5.** m values for the quantified damage.

**m**	**value**

Bearing	3.33
Ring Gear Bending	6.3
TM Gear Bending	8.389
TM Gear Surface (Case)	17.857
